# A study on the effectiveness of N95 mask-wearing training through fit tests for healthcare workers at a tertiary hospital in South Korea

**DOI:** 10.1017/ash.2025.118

**Published:** 2025-09-03

**Authors:** Mi-Jin Hong, Ji-Young Lee

**Affiliations:** Infection Control TeamCatholic University Seoul St. Mary’s Hospital

**Keywords:** N95 mask, Healthcare workers(HCWs), Training, Quantitative fit test(QNFT)

## Abstract

**Background:** The risk of respiratory infection varies on the degree of the fit of N95 masks, so education and training of appropriate wearing methods are required. This study was conducted to investigate whether there are differences in the fit of N95 masks among healthcare workers(HCWs) with education and experience in N95 mask-wearing and to assess the effectiveness of N95 mask-wearing training through fit tests. **Methods:** From October 2023 to February 2024, training on the wearing of N95 masks was conducted through fit tests for 195 high-risk department HCWs and new HCWs at a tertiary hospital. Fit tests was conducted before and after the training. Previous experiences of N95 mask-wearing education were investigated using questionnaires. The fit test was measured using QNFT (Quantitative Fit Test). Data was analyzed using percentages and a chi-square test. **Result:** Out of the 195 participants, 44 HCWs had experience by group or rote learning. The fit test pass rate in the group with education experience was 45.5%, which was higher than the 32.9% in the group without education experience; however, there was no statistically significant difference (P=0.293). The fit test pass rate for N95 mask-wearing training increased significantly from 35.8% (70 HCWs) before training to 98.5% (192 HCWs) after training (p=0.000). The three HCWs who failed the first test all passed the fit test after retraining using N95 masks of different shapes and sizes. **Discussion:** It was confirmed that N95 mask-wearing training through fit tests was effective in increasing fit, whereas group or rote learning was not effective. N95 mask-wearing training through fit tests is an effective method to enhance N95 mask fitting. It is essential to explore diverse approaches to sustain the training impact.

